# Insights on the Functional Impact of MicroRNAs Present in Autism-Associated Copy Number Variants

**DOI:** 10.1371/journal.pone.0056781

**Published:** 2013-02-25

**Authors:** Varadarajan Vaishnavi, Mayakannan Manikandan, Basant K. Tiwary, Arasambattu Kannan Munirajan

**Affiliations:** 1 Department of Genetics, Dr. ALM PG Institute of Basic Medical Sciences, University of Madras, Chennai, Tamil Nadu, India; 2 Centre for Bioinformatics, School of Life Sciences, Pondicherry University, Pondicherry, India; CNRS UMR7275, France

## Abstract

Autism spectrum disorder is a complex neurodevelopmental disorder that appears during the first three years of infancy and lasts throughout a person’s life. Recently a large category of genomic structural variants, denoted as copy number variants (CNVs), were established to be a major contributor of the pathophysiology of autism. To date almost all studies have focussed only on the genes present in the CNV loci, but the impact of non-coding regulatory microRNAs (miRNAs) present in these regions remain largely unexplored. Hence we attempted to elucidate the biological and functional significance of miRNAs present in autism-associated CNV loci and their target genes by using a series of computational tools. We demonstrate that nearly 11% of the CNV loci harbor miRNAs and a few of these miRNAs were previously reported to be associated with autism. A systematic analysis of the CNV-miRNAs based on their interactions with the target genes enabled the identification of top 10 miRNAs namely hsa-miR-590-3p, hsa-miR-944, hsa-miR-570, hsa-miR-34a, hsa-miR-124, hsa-miR-548f, hsa-miR-429, hsa-miR-200b, hsa-miR-195 and hsa-miR-497 as hub molecules. Further, the CNV-miRNAs formed a regulatory loop with transcription factors and their downstream target genes, and annotation of these target genes indicated their functional involvement in neurodevelopment and synapse. Moreover, miRNAs present in deleted and duplicated CNV loci may explain the difference in dosage of the crucial genes controlled by them. These CNV-miRNAs can also impair the global processing and biogenesis of all miRNAs by targeting key molecules in the miRNA pathway. To our knowledge, this is the first report to highlight the significance of CNV-microRNAs and their target genes to contribute towards the genetic heterogeneity and phenotypic variability of autism.

## Introduction

Autism Spectrum Disorder (ASD) refers to a group of heterogeneous neurodevelopmental disorders characterized by impairments in communication, social interaction and restricted, repetitive and stereotypic patterns of behavior [1]. The severity of impairment varies from individual to individual ranging from mild to profound, with approximately 40% of affected individuals exhibiting a significant cognitive deficit [2]. Current estimates indicate that ASD have a median prevalence of 62 per 10,000 children [3]. The global burden of autism continues to increase urging the need for studying the underlying principles of neuronal and behavioral changes observed in the affected individuals. The strong genetic basis of this disorder is evident from the high concordance rate observed in monozygotic twins than in dizygotic twins (>90% vs. 10%) [4], [5]. In spite of the substantial heritability, identification of genetic variants and specific genes associated with ASD remains challenging. A pioneering study by Sebat et al., (2007) and The Autism Genome Project Consortium showed that a particular type of structural polymorphism denoted as ‘copy number variations’ (CNVs) in the form of microdeletions and microduplications at multiple chromosomal loci spanning several hundreds of nucleotides to megabases were implicated in autism. Among these CNVs several were recurrent as previously observed in autistic cases and a few were identified at new genomic locations including 2p16, 1q21 and 17p12 [6], [7], [8], [9]. Subsequent studies have firmly implicated CNVs at 16p11.2, 7q11.23, 15q11–13, 22q11.2 and 1q21.1 in ASD indicating their importance [10], [11], [12], [13]. A striking observation was the high prevalence of *de novo* CNVs (i.e. CNVs not present in either parent) in both sporadic and familial cases of ASD compared with controls, and were often detected at loci enriched for genes such as *SHANK3*, *NRXN1* and the neuroglins that are known to regulate synaptic differentiation and glutaminergic neurotransmitter release [14]. Together, these findings have established CNVs as a substantial risk factor for development of ASD.

Emerging evidences point out that certain CNVs may disrupt the homeostasis of neuronal development resulting in a range of disorders as part of a neurodevelopmental continuum. CNVs can have great phenotypic impact by altering gene dosage, disrupting coding sequence, or deregulating gene expression [15]. Gene expression levels can be either positively correlated [16], [17] or negatively correlated [18] with copy number increment. Depending on the gain or loss of dosage-sensitive genes, CNVs can have both beneficiary and detrimental impact and may confer genetic predisposition to diseases [19], [20]. To date, almost all CNV studies in autism have concentrated only on the genes, leaving the important non-coding regulatory microRNAs (miRNAs) present in these regions. MicroRNAs, a class of small (∼21 nt) single-stranded RNAs particularly abundant in the brain, are known to regulate gene expression at the transcript level through RNA-induced silencing complex (RISC) mediated translational inhibition or very rarely through mRNA cleavage by binding to the 3′ untranslated region (3′UTR) of target mRNAs [21]. Dysfunction of neuronal miRNAs may result in an array of neuropathological conditions (reviewed in [22]) and it is reported that neural miRNAs and their target mRNAs are co-expressed, suggesting their participation in feedback and feed forward mechanisms that connect global transcriptional activation with the control of local dendritic protein synthesis [23]. Interestingly, the functions attributed to miRNAs overlap with the growth abnormalities [24], delays and disorganization in neuronal maturation [25] in the brain of autistic individuals. Aberrations in the translational control of multiple mRNA targets mediated by each miRNA could lead to the difference in phenotypes observed in ASD. Conversely, multiple miRNAs can target the same mRNA leading to convergent phenotypes arising from various CNV loci. Based on the above considerations, it is clear that characterizing miRNAs residing at the CNV loci may illustrate the complexity underlying neuronal development, function and dysfunction which will eventually help in understanding and improving the effective management of autism. Hence, in this study we followed a stepwise approach to investigate how miRNAs present in the CNV regions contribute to autism.

## Results

### Identification and Analysis of microRNAs Present in Autism-associated CNV Loci

**Figure 1:**
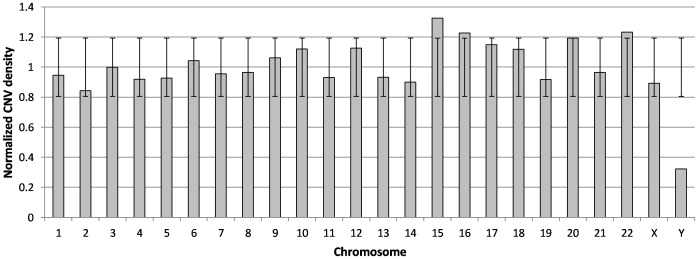
Normalized density distribution of autism-associated CNVs across human chromosomes. The normalized CNV density is plotted in y-axis against the chromosomes in x-axis. For any given chromosome, a value above 1 indicates that it has accumulated more number of autism-associated CNVs compared to the mean of all chromosomal CNV densities. The error bars indicate the standard deviation.

**Figure 2:**
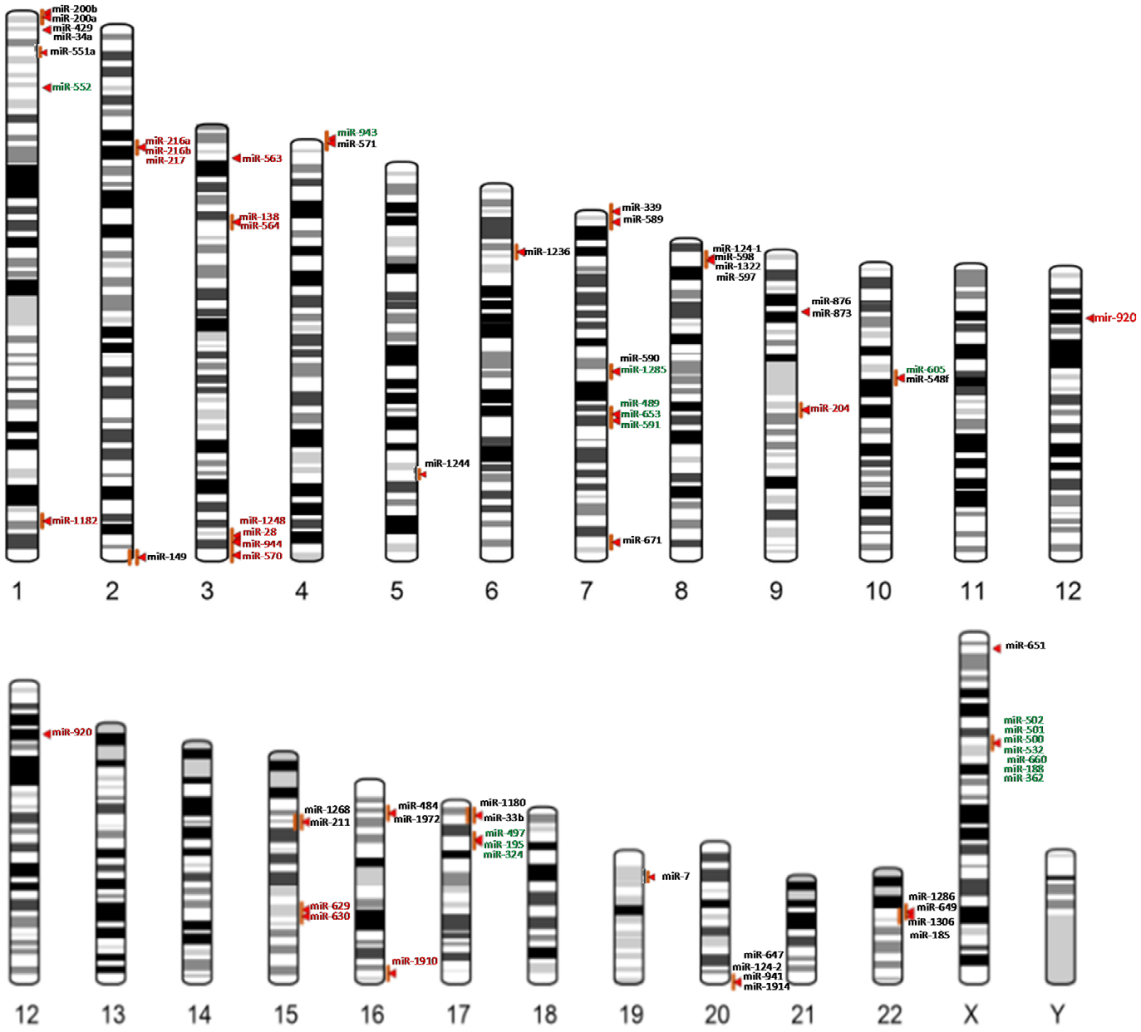
The genomic location of autism-associated CNV-microRNAs. The genomic locations of miRNAs present in the 41 CNV loci consistently associated with autism are indicated with arrow heads. The miRNAs labelled in red, green and black indicate the deleted, duplicated and deleted-duplicated categories, respectively.

From Autism Database (AutDB) [26], we retrieved 1145 CNVs that were classified based on their type (i.e., deletion/duplication), chromosomal loci and number of association studies. A rigorous analysis of these CNVs suggested that almost the entire human genome is implicated in autism. In order to identify the ‘hot-spot’ chromosomes that have accumulated many autism-associated CNVs, we calculated the normalized CNV density across the human genome. Since the length of the chromosomes can impose bias on the total number of CNVs that it harbors, we corrected (i.e., normalized) the density values and the results are presented in Figure 1. Chromosomes 15, 16 and 22 were significantly enriched for CNVs. Although chromosomes 6, 9, 10, 12, 17, 18 and 20 had a normalized CNV density value above 1, the enrichment is unlikely to be significant since the density scores were within one standard deviation of the mean of all densities. This substantially supports the established fact that CNVs are collectively common on the whole, but extremely rare for any specific loci (<1%). Further, Y chromosome is a clear outlier in the CNV density data. This could be explained by the association of Y chromosome CNVs with infertility thereby eliminating inheritance of existing CNVs and lack of homologous chromosome for generating *de novo* CNVs [27], [28]. Next we searched for CNVs that are consistently reported to be associated with autism by different research groups and analysed the coordinates of 378 CNVs falling within our selection criteria (see materials and methods) in UCSC Genome Browser – hg18, for the presence of miRNAs. This resulted in the identification of 41 CNV loci (11 deletions, 9 duplications and 21 combinations) harboring 71 miRNAs including isomiRs (Figure 2). Further only a small proportion of the miRNAs present in CNV regions that showed deletion as well as duplication in different individuals overlapped with the exclusively deleted or duplicated miRNAs. The CNV loci, their miRNA content and the number of association studies reporting that particular CNV in autism are listed in Table S1. Out of the 71 CNV associated miRNAs, hsa-miR-484, hsa-miR-598, hsa-miR-7, hsa-miR-195, and hsa-miR-211 were previously reported to be associated with autism [29], [30], [31]. MicroRNA-34a [32], [33], hsa-miR-188 [34] and hsa-miR-124 [35] were known to have neuronal functions suggesting that miRNAs present in autism-associated CNVs might be critical determinants or contributors to disease phenotype.

### Integrating miRNAs and their Autism Associated Target Genes into a Regulatory Network

**Figure 3:**
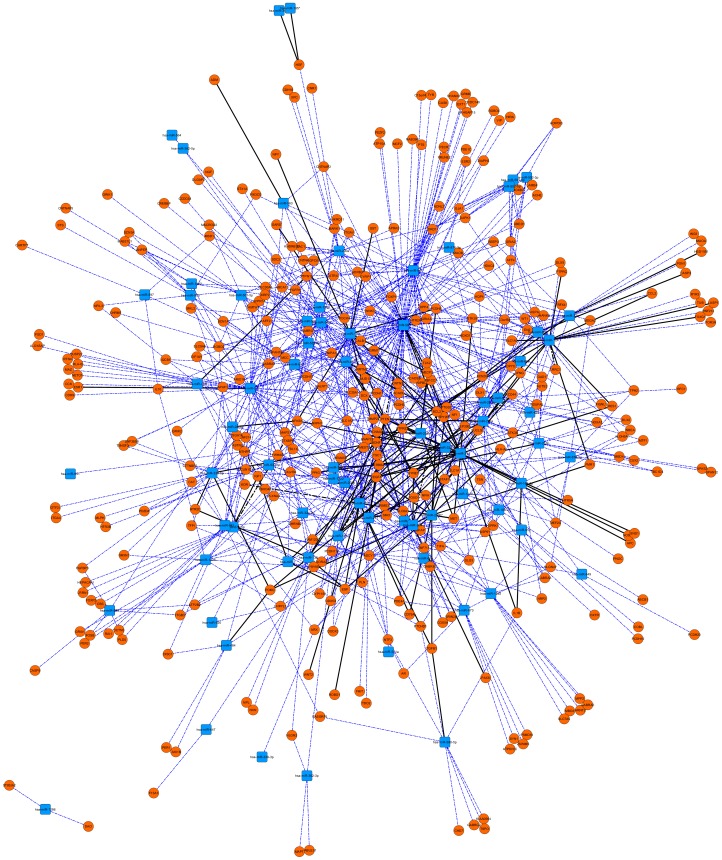
CNV-microRNA-target genes interaction network in autism. Saffron ellipses represent the target genes of miRNAs present in the CNV loci, while miRNAs are denoted by the blue coloured squares. Dashed blue lines represent the predicted interactions and solid black edges represent the validated interactions.

To explore the molecular functions of genes targeted by these miRNAs, we retrieved their validated and putative targets from miRWalk [36], a comprehensive database that provides information on predicted as well as validated binding sites of miRNAs on their target genes with options to choose targets cross-predicted by 8 established miRNA-target prediction programs. From all the target genes, the autism-related targets were identified through comparison with a list of 708 genes known to be implicated in autism (see materials and methods section for details). The resulting dataset comprising the known regulatory interactions as well as the predicted regulatory interactions between miRNAs and mRNAs of target genes was visualized using Cytoscape^©^ 2.8.3 [37] (Figure 3). This bipartite network contained 400 nodes (miRNAs and target genes) connected by 926 edges (interactions). It is evident that except hsa-miR-1286 and its two targets *ST8SIA2* and *DAO*, the whole network has a strongly connected component. At the level of whole networks, measures that are often used to identify critical molecules called ‘hubs’ are the degree and degree distribution [38]. The degree is defined as the connectivity of individual nodes i.e., the number of interactions. Comprehensive analysis of the miRNA–target gene network indicated that a few nodes exhibited a high degree value in accordance with the power law [38]. We defined the top 10 nodes with the highest degree values as hubs (Table 1) that act as converging centres which are often essential, indicating their overall importance in the miRNA-gene regulatory network. All the hub miRNAs except hsa-miR-548f were known to be associated with neuronal disorders. The miRNAs hsa-miR-591, hsa-miR-598, hsa-miR-1180, hsa-miR-1268, hsa-miR-1306, hsa-miR-1910 and hsa-miR-1914 were not included in the network as they did not meet the target gene prediction criterion.

**Table 1:** The top 10 hub molecules in the autism CNV-microRNA-target gene network.

MicroRNAs	Description	Implications in autism or other neuronaldisorders (PubMed ID)	Notable targets*	Degree
hsa-miR-590-3p	Mature miRNA originatingfrom 3′ end of *Homo* *sapiens* miR-590stem-loop	Alzheimer disease(21548758)	*PTEN, CNTNAP2, IGF1, FEZF2, BCL2, GRIA2, EP300,* *SEMA5A, KCNMA1, GABRG1, SLC6A1, NF1, ATRX, ERBB4,* *FOXP1, NPY2R, GABRA4, SHANK2, MECP2, GRIA3,* *NDNL2, PCDH9, WT1*	91
hsa-miR-944	*Homo sapiens* miR-944stem-loop	No known association with neuronal disorders.Involved in cancer metastasis to brain (22331473)	*GRIA2, GABRG1, NF1, ESR2, GRM7, GRM8, SHANK3,* *GRIA3, VEGFA, FOXP1, NDNL2, GABRA4, ATRX,* *ARHGAP15, UBE2H, DCX*	50
hsa-miR-570	*Homo sapiens* miR-570stem-loop	Pituitary tumorigenesis, pituitary adenomacells (22564666)	*PTEN, GRM7, TBL1X, ATRX, SLC1A2, SCN1A, ERBB4,* *MAZ, FOXO1, GRIA3, ESR1, SLC9A6, CDH9*	46
hsa-miR-34a	*Homo sapiens* miR-34astem-loop	Schizophrenia (21738743), Brain Neoplasms(20190569), Bipolar disorder (20675101)	*PTEN, NFKB, VEGFA, KCNMA1, FOXO1, HES1, ERBB4,* *BCL2, SYNE1, GABRA3, FOXP1, MET*	32
hsa-miR-124	*Homo sapiens* miR-124stem-loop	Autism (19204725), Alzheimer Disease(22178568),Cerebral Infarction(21551949), Fragile×Syndrome(18687414)	*BDNF, HES1, SLC1A3, FOXG1, SHANK2, SYNE1,* *GRIA3, FMR1, GRIA2, PTPRC*	31
hsa-miR-548f	*Homo sapiens* miR-548fstem-loop	No known association with neuronal disorders	*PTEN, SLC1A1, GRIK2, GABRG1, SLC1A3, NGF, GABRA4*	31
hsa-miR-429	*Homo sapiens* miR-429stem-loop	Drug addiction (22436491), non-learnedhelplessness in rats (21275079)	*EGF, PTEN, MET, SEMA5A, SLC6A1, SLC1A2, ERBB4,* *NPY2R, EP300, NTF3, TGFB1*	28
hsa-miR-200b	*Homo sapiens* miR-200bstem-loop	Drug addiction (22436491), non-learnedhelplessness in rats (21275079)	*SLC6A1, SLC1A2, SEMA5A, NPY2R, ERBB4, NTF3*	23
hsa-miR-195	*Homo sapiens* miR-195stem-loop	Autism (20374639)Schizophrenia (17326821)	*PTEN, NPY, SLC1A2, BDNF, BCL2, MECP2,* *PCDH8, MTHFR*	23
hsa-miR-497	*Homo sapiens* miR-497stem-loop	Regulates neuronal death in mouse brain(20053374), involved in neurogenesis (21677392)	*SLC1A2, SLC9A6, TSC1, PTEN, BCL2, KCNMA1, FGFR,* *MTHFR, PCDH9, SYNE1*	23

* Only selected targets are shown. For the complete list of target genes, see supporting information Table S5.

### Analysis of Global Properties of the Network

A potential source of bias in networks is that disease causing molecules may have higher degrees simply because they were better studied. Therefore, to confirm the association of CNV-miRNAs with autism, we constructed another network in a similar manner with equal number of random non-CNV miRNAs. A comparison of local network properties of the two networks using Two-sample Kolmogorov-Smirnov test indicated that the autism CNV-miRNA target gene network differs significantly from the non-CNV miRNA target gene network by 5 properties namely average shortest path (D = 0.1374, *P* = 0.0008472), closeness centrality (D = 0.1374, *P* = 0.0008472), eccentricity (D = 0.1119, *P* = 0.01156), neighbourhood connectivity (D = 0.1467, *P* = 0.0002826) and radiality (D = 0.1374, *P* = 0.0008472). The remaining local properties namely betweenness centrality, degree, number of directed edges, stress and topological coefficient were identical between the two networks. Further, we removed the top 10 hub miRNAs one at a time from the autism CNV-miRNA regulatory network and then analysed the network properties using NetworkAnalyzer, a Java plug-in for Cytoscape. The diameter, characteristic path length and density of the network remained unchanged after removal of the hub molecules indicating the robustness of the network. A t-test for Poisson counts was applied to infer the statistical significance which showed that there was a significant increase in connected components after removal of hub 1 (*P = *0.00136), hub 2 (*P = *0.00227), hub 3 (*P = *0.01049) and hub 5 (*P = *0.02894). The number of isolated nodes increased significantly after removal of hub 1 (*P = *0.00027), hub 2 (*P = *0.00046), hub 3 (*P = *0.00235), hub 4 (*P = *0.0228) and hub 5 (*P = *0.00718). Taken together, hub 1 (hsa-miR-590-3p) plays a very crucial role in the network. It is worthy to mention that hsa-miR-124 which is already known to have broad neuronal functions lies within the top 5 hubs in the network.

### Annotating the Biological Functions of microRNA Target Genes

Elucidating how microRNAs operate at molecular, cellular and hierarchical (pathway) levels may provide novel insights on the neuropathophysiology of autism. Hence we annotated the functions of the 326 autism-associated gene targets of miRNAs (Table 2) and found that they were well in compliance with the functional annotation of the total 708 genes implicated in autism (Table S2). In brief, the genes were involved in transmission of nerve impulse, behavior, central nervous system development, postsynaptic membrane part, axon part, GABA type A receptor activity and glutamate receptor activity. Neuroactive ligand receptor interaction pathway exhibited the highest fold-enrichment score in the KEGG pathway enrichment analysis (R = 14.71, *P = *2.29×10^−21^) suggesting that perturbations of these genes by miRNAs may alter the spectrum of neurological functions resulting in autistic phenotype. To test whether the neuronal-related functions were specific to the autism-associated gene set, we annotated the baseline functions of all target genes of CNV-miRNAs. The annotation results indeed confirmed the neuronal specificity of the autism-associated target genes, since the collective annotation assigned generalized functions such as metabolic and cellular processes (Table S3). A better control for this functional annotation would be to choose target genes of miRNAs present in CNVs of healthy individuals. Although, a number of databases have documented control CNVs, we were afraid of choosing specific CNVs as this will introduce manual bias and may not truly represent the procedure of selecting the autism-related CNVs that harbor miRNAs. Hence we annotated the functions of all the targets of CNV-miRNAs, and established that only the autism-related genes were enriched for neurobiological functions.

**Table 2:** Functional annotation of the 326 genes targeted by miRNAs present in autism-associated CNV loci.

GO category	R	adj P
***Biological process***		
Transmission of nerve impulse – GO:0019226	7.11	2.69×10^−29^
Synaptic transmission – GO:0007268	7.07	7.12×10^−25^
Nervous system development – GO:0007399	3.91	1.30×10^−24^
Cell-cell signaling – GO:0007267	4.78	3.37×10^−24^
Behavior – GO:0007610	5.77	3.48×10^−23^
System development – GO:0048731	2.55	3.64×10^−22^
Anatomical structure development –GO:0048856	2.40	2.16×10^−20^
Regulation of neurological system process –GO:0031644	10.72	1.30×10^−19^
Central nervous system development –GO:0007417	5.68	2.66×10^−19^
Multicellular organismal process –GO:0032501	1.88	4.41×10^−19^
***Molecular function***		
Protein binding – GO:0005515	1.36	1.96×10^−09^
Extracellular ligand-gated ion channel activity –GO:0005230	9.69	2.20×10^−08^
Ligand-gated ion channel activity –GO:0015276	5.97	2.03×10^−06^
Ligand-gated channel activity – GO:0022834	5.97	2.03×10^−06^
Amine binding – GO:0043176	6.49	2.03×10^−06^
Glutamate receptor activity – GO:0008066	13.94	3.46×10^−06^
Protein dimerization activity – GO:0046983	2.85	1.89×10^−05^
Ionotropic glutamate receptor activity –GO:0004970	16.85	3.12×10^−05^
Binding – GO:0005488	1.12	3.12×10^−05^
GABA-A receptor activity – GO:0004890	15.96	3.68×10^−05^
***Cellular component***		
Neuron projection – GO:0043005	5.76	9.40×10^−15^
Synapse – GO:0045202	5.46	9.40×10^−15^
Postsynaptic membrane – GO:0045211	8.92	7.14×10^−14^
Synapse part – GO:0044456	6.27	1.45×10^−13^
Axon – GO:0030424	8.14	3.42×10^−13^
Cell projection – GO:0042995	3.45	4.07×10^−11^
Cell soma – GO:0043025	6.76	5.01×10^−10^
Plasma membrane part – GO:0044459	2.13	1.15×10^−09^
Cell junction – GO:0030054	3.42	1.74×10^−08^
Axon part – GO:0033267	12.00	2.70×10^−08^
***KEGG pathway***		
Neuroactive ligand-receptor interaction –04080	14.71	2.29×10^−21^
Pathways in cancer –05200	10.57	1.60×10^−16^
Prostate cancer –05215	18.81	7.41×10^−11^
Calcium signaling pathway –04020	11.76	1.04×10^−10^
Amyotrophic lateral sclerosis (ALS) –05014	23.69	3.06×10^−09^
Small cell lung cancer –05222	16.61	9.16×10^−09^
Renal cell carcinoma –05211	17.94	2.89×10^−08^
Focal adhesion –04510	9.02	3.75×10^−08^
Metabolic pathways –01100	3.41	4.58×10^−07^
MAPK signaling pathway –04010	6.74	9.49×10^−07^

The results for each enriched GO categories of the 326 genes targeted by the autism associated CNV-miRNAs are listed in this table. Each GO category belongs to one of the three sub-roots (*biological process, molecular function, or cellular component*). R is the ratio of enrichment. Adj P – P-value adjusted by multiple testing.

### MicroRNAs and their Target Genes can Contribute to the Genetic and Phenotypic Heterogeneity of Autism

Considering the negative regulation of their target genes by miRNAs and the strong association of CNVs to autism together with genetic heterogeneity, we reasoned that a CNV-miRNA would display an inverse correlation with its targets, if the targets are also CNV-associated. In other words, if a CNV loci harboring the miRNA shows deletion, there is an equal chance for the CNV loci harboring its respective or crucial target gene to show duplication in yet another individual as the final outcome would be the same. However, a deletion of a single miRNA would produce a more severe autistic phenotype due to its pleiotropic effect rather than the deletion of a smaller subset of target genes. To test the above points, we utilized the individual data available in AutDB and analysed the target genes present within the CNV loci for any relationship with their corresponding miRNA. We limited our analysis with the top 10 hub miRNAs and their target genes that were enriched in the functional categories of transmission of nerve impulse, synaptic transmission and central nervous system development to obtain a high confidence dataset. As expected, an inverse correlation was observed in a sub-population of miRNAs and their targets genes (Table 3). Besides, a single miRNA can deregulate all of its downstream target genes irrespective of whether they are located within or outside the CNV loci, thereby leading to a combinatorial decrease/increase in their expression that may account for the disease phenotype. It is plausible that in the autistic population it is the lack of functional redundancy that could account for the origin of the syndrome. As rightly pointed out by one of our reviewers, it would also be interesting to investigate the miRNA gene targets that lie outside the CNV regions in autistic individuals vs. control individuals which could also be the underlying cause of ASD. Even a partial loss of function of the miRNA targets could contribute to the pathobiology of autism. For example, haploinsufficiency of *Shank3*, *CMIP*, *FOXP2* and *Tsc2* has already been linked to the etiology and phenotypic characteristics of autism [39], [40], [41], [42]. Taken together, the CNV-associated miRNAs and their target genes may explain in part the genetic and phenotypic complexity observed in ASD individuals harboring these structural variations.

**Table 3:** Inverse correlation between the top 10 hub CNV-miRNAs and their target genes.

Deleted MicroRNA: Patient ID (AutDB Reference)	Duplicated target Gene: Patient ID (AutDB Reference)
**miR-590**: 14659, 22908 & 22928 (Rosenfeld JA 2010)	**ACHE**: 11415 & 11524 (Celestino-Soper PB 2011), 5411_3 (Pinto D 2010)**ATRX**: 6379_4 (Pinto D 2010)**CNTN4**: subject1 (Roohi J 2009), Owely_E016, AU077105, AU082803, AU0913301, AU0913302, AU1070302, AU1407301, AU1427302 & AU1427303 (Glessner JT 2009), 5269_3 (Pinto D 2010)**CNTNAP2**: 3018_003, 5006_3, 5368_3, 6266_3 & 6320_4 (Pinto D 2010)**GAD1**: 5320_3 (Pinto D 2010)**GRIA2**: HI3912 (Itsara A 2010)**NTF3**: Case 22 & Case 35 (Bremer A 2011)**MECP2**: Case 12 (Bremer A 2011), 6164_4 (Pinto D 2010), 23009 (Rosenfeld JA 2010)
**miR-570, miR-944**: Case 2 (Bremer A 2011)	**ATRX**: 6379_4 (Pinto D 2010)
**miR-570**: Case 2 (Bremer A 2011)	**HTR2C**: SK0088-003 (Marshall CR 2008)
**miR-944**: Case 2 (Bremer A 2011)	**GRIA2**: HI3912 (Itsara A 2010)
**miR-124-1**: NAAR007-E6-8175-201 (Autism Genome Project Consortium 2007)	**BDNF**: patient1 & patient2 (Shinawi M 2011)
**miR-124-3**: HI2592 & HI2991 (Itsara A 2010)	**GRIA2**: HI3912 (Itsara A 2010)**CCL2**: SK0284-003 (Marshall CR 2008)
**miR-563**: Case 3 (Qiao Y 2009)	**CNTN4**: subject1 (Roohi J 2009), Owely_E016, AU077105, AU082803, AU0913301, AU0913302, AU1070302, AU1407301, AU1427302 & AU1427303 (Glessner JT 2009), 5269_3 (Pinto D 2010)**CNTNAP2**: 3018_003, 5006_3, 5368_3, 6266_3 & 6320_4 (Pinto D 2010)
**miR-653**: Case 1 (Dauwerse JG 2010)	**CNTN4**: subject1 (Roohi J 2009), Owely_E016, AU077105, AU082803, AU0913301, AU0913302, AU1070302, AU1407301, AU1427302 & AU1427303 (Glessner JT 2009), 5269_3 (Pinto D 2010)
**miR-1285**: Case 1 (Dauwerse JG 2010)	**GABRA5, GABRB3**: 12007.p1 (Sanders SJ 2011)
**miR-629**: 11233.p1 (Levy D 2011), AU008 (McInnes LA 2010), 11233.p1 (Sanders SJ 2011)	**GABRB3**: 12007.p1 (Sanders SJ 2011)
**miR-34a**: 5065_3, 5099_3, 5106_3, 5215_3, 5218_3, 5241_3 & 5319_3 (Pinto D 2010)	**FOXO1**: 12343.p1 (Sanders SJ 2011)
**Duplicated MicroRNA: Patient ID (AutDB Reference)**	**Deleted target Gene: Patient ID (AutDB Reference)**
**miR-590**: patient6, patient7 (Dixit A 2012), 11129.p1(Levy D 2011), subjectA (Malenfant P 2012), Case 4(Qiao Y 2009), 17880, 20963 & 28233 (Rosenfeld JA 2010),11129.p1 & 11154.p1 (Sanders SJ 2011)	**ACHE**: 11076, 11178 & 11271 (Celestino-Soper PB 2011), 1960_301 (Pinto D 2010)**CNTN4**: subject2B & Subject2C (Roohi J 2009), AU066005 (Christian SL 2008), AU066004 & AU066005 (Gai×2012)**CNTNAP2**: Case 29 (Bremer A 2011), AU038303 (Davis LK 2009)**ERBB4**: Case 19 (Bremer A 2011)
**miR-124-3**: HI1892 & HI4468 (Itsara A 2010), MM0109-003(Marshall CR 2008)	**FOXG1**: Case 1 (Jacob FD 2009)**HDAC4**: 4314 (Rosenfeld JA 2010), Van69-258900 (Sebat J 2007)**HES1**: 17184 (Rosenfeld JA 2010)**SUCLG2**: AU003404 (Christian SL 2008), NAAR007-B4-AU32503, NAAR007-B6-8159-203 & NAAR007-B2-8159-101 (Autism Genome Project Consortium 2007)
**miR-195**: 11532.p1 (Levy D 2011)	**BDNF**: patient1 & patient2 (Shinawi M 2011)
**miR-34a**: 5408_3 (Pinto D 2010)	**ERBB4**: Case 19 (Bremer A 2011)**HES1**: 17184 (Rosenfeld JA 2010)**FOXP1**: patientA (Hamdan FF 2010)
**miR-571**: 13367.p1 (Sanders SJ 2011)	**ERBB4**: Case 19 (Bremer A 2011)
**miR-188-5p**: proband (Chung BH 2011), SK0306-004(Marshall CR 2008)	**MEF2C**: patient2 (Shinawi M 2011)
**miR-497**: 11532.p1 (Levy D 2011)	**STX1A**: 14659, 22908 & 22928 (Rosenfeld JA 2010)

This table was compiled after comprehensive analysis of the individual data available in AutDB. For convenience and to gain a high confidence dataset we analysed only the top 10 hub miRNAs and their target genes that were enriched in the functional categories transmission of nerve impulse, synaptic transmission and central nervous system development and hence this list is not exhaustive. The miRNA-gene pairs that did not show inverse correlation were excluded.

### The Dosage of microRNAs and Target Genes

**Figure 4:**
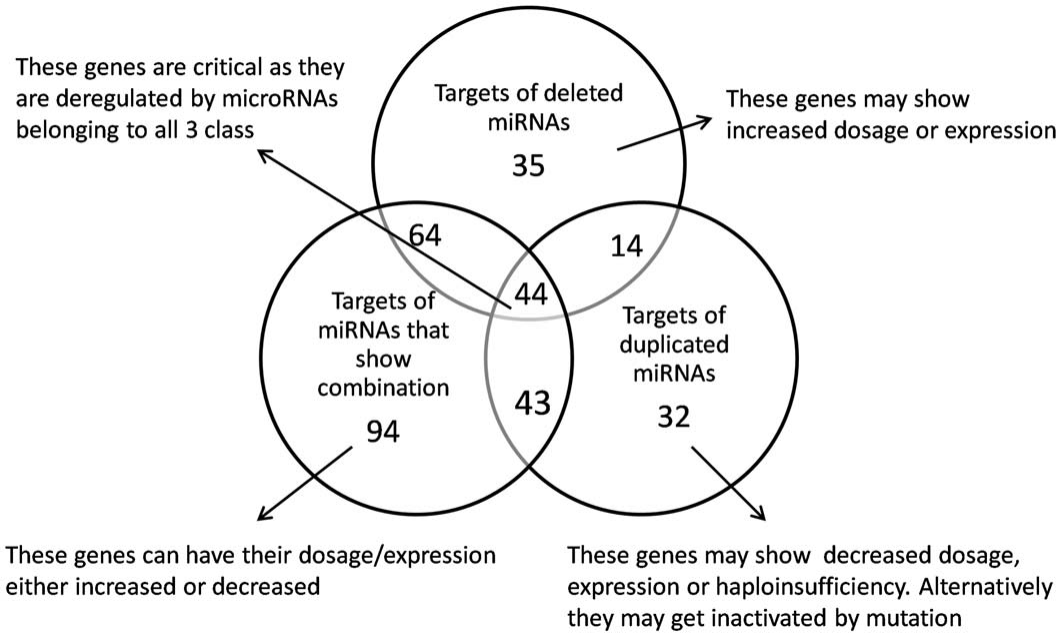
Intersection analysis of the 326 genes targeted by miRNAs present in CNVs. The Venn diagram represents the distribution of genes targeted by microRNAs belonging to three CNV categories (deletion, duplication and deletion-duplication). The effect on the dosage/expression status of these genes is described along the sides of the figure with arrow mark indications.

As structural variations are large events, they can have a profound impact on the dosage of many genes suggesting that abnormal gene dosage or expression might play a key role in pathogenesis or susceptibility to ASD. It has been presumed that the expression of CNV genes/miRNAs would correlate with copy number and so a gene/miRNA that has one allele duplicated (copy number of 3) would have 1.5 times the level of expression of the wild type copy number of 2. A change in miRNA expression or dosage will affect the expression of all of its target genes and may have a pleiotropic effect. Hence the target genes of miRNAs were subjected to intersection analysis to identify genes whose expression can be potentially altered (Figure 4). The results clearly indicate that 35 genes were exclusively targeted by the deleted miRNAs and hence may show an increase in dosage or expression, while 32 genes were exclusively targeted by duplicated miRNAs and may show decreased expression. The expression of 94 genes targeted by miRNAs present in the CNV loci that showed either deletion or duplication in different individuals can fluctuate from low to high. We do not expect all the genes in each of the above mentioned categories to have an observable difference in dosage/expression. However, a subset in each category might be critical and future experimental studies may reveal their importance (For the complete list of genes in each category, refer Table S4). Further, 44 genes were found to be deregulated by miRNAs belonging to all three categories suggesting their central role and important functional contribution to autism. Analysis of these 44 genes revealed that this cluster contains critical genes. In brief, *OPRK1*, *GRIA2*, *GABRA4* and *GRIA3* are important components of the neuroactive ligand-receptor interaction pathway; *BCL2*, *BDNF* and *NFKB1* belong to the neurotrophin signalling pathway; *GRIA2* and *ADCY1* are involved in long-term potentiation with the additional role of *GRIA2* in long-term depression together with *GRIA3*. The genes *BCL2*, *GRIA2* and *SLC1A2* are also components of Amyotrophic Lateral Sclerosis (ALS) pathway.

### MicroRNAs Regulate Transcription Factors that Govern the Expression of Autism-related Genes

**Figure 5:**
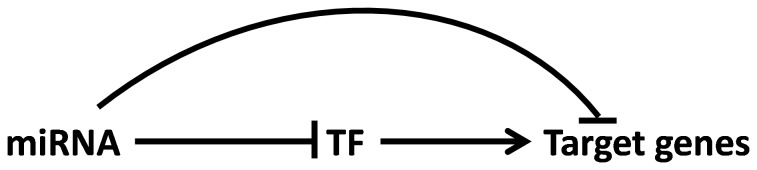
Different modes of repression of target genes caused by miRNAs. A miRNA can directly repress its target genes or can repress the TF that governs the expression of the miRNA’s targets. In addition, the miRNA can repress the TF as well as its target genes thereby having dual effect on repression of the downstream target genes.

MicroRNAs are well known to mediate feedback loops and feed forward systems [23] and a few reports suggest that transcription factors (TFs) are clustered at miRNA network hubs [43], [44]. The relationship between TFs and miRNAs that act in a coordinated fashion in biological or disease contexts can presumably help in defining regulatory controls. Further, a miRNA can have double the effect on the expression of its target genes when it regulates as well the TF that governs the expression of the genes targeted by miRNAs as represented in Figure 5. We tested whether autism CNV-miRNAs directly regulate the expression of the TFs that control the expression of the target genes. Chip enrichment analysis (ChEA) of the 326 target genes indicated that nearly 64% of them (208/326) were under the control of the 15 TFs presented in Table 4. Our analysis revealed that miRNAs present in autism-associated CNV loci can potentially regulate the spatial and temporal dynamics of neuronal genes and thus have an important role in determining the genetic heterogeneity and neurobiological changes attributed to autism. Although a number of studies have focused on miRNA function in neuronal circuits, mechanistic insights require analysis of individual miRNAs, TFs and the target genes they control.

**Table 4:** Regulatory loop between transcription factors, CNV-associated miRNAs and their target genes.

Transcriptionfactor-ChEA ID	*P*-value	CNV-miRNAs regulating the TF	No. of autism-associated genes under the control of the corresponding TF
POU3F2-20337985	2.42×10^−9^	miR-204, miR-552, miR-590-3p	53
MYC-20876797	7.82×10^−9^	miR-200b &200b*, miR-195&195*, miR-7, miR-34a &34a*,miR-200a&200a*, miR-149&149*, miR-429, miR-138	46
TP53-18474530	4.71×10^−6^	miR-605, miR-33b&33b*, miR-200b &200b*, miR-34a &34a*,miR-200a &200a*, miR-149&149*, miR-429, miR-28-5p&3p,miR-630, miR-1285, miR-204, miR-217, miR-216a,miR-324-5p, miR-195&195*, miR-497&497*	28
RUNX1-17652178	8.46×10^−6^	Cross-comparison did not give any result.	31
POU5F1-16153702	7.91×10^−5^	Not predicted by miRWalk	21
SALL4-22934838	2.00×10^−4^	miR-497, miR-195	35
SOX2-16153702	2.95×10^−4^	miR-200b, miR-590-5p, miR-429, miR-1914&1914*, miR-204,miR-1910	32
E2F1-17053090	4.77×10^−4^	miR-362-3p, miR-570, miR-34a &34a*, miR-149&149*	39
NANOG-16153702	5.94×10^−4^	Not predicted by miRWalk	38
TFAP2A-17053090	8.63×10^−4^	Criteria not met (Predicted targets were concordant onlyamong 3 databases instead of 4).	41
TCF4-18268006	0.00371	miR-204, miR-629, miR-200a, miR-590-3p, miR-211	14
ESR1-20079471	0.00801	miR-570	8
ERG-21242973	0.00999	Cross-comparison did not give any result.	10
WT1-19549856	0.01637	miR-216a, miR-590-3p	7
MYC-18940864	0.0348	miR-200b &200b*, miR-195&195*, miR-7, miR-34a &34a*,miR-200a&200a*, miR-149&149*, miR-429, miR-138	16

This table was compiled after analysis of data retrieved from ChEA and miRWalk database. Only transcription factors enriched with a statistically significant *P*-value score of <0.05 are shown. The validated microRNAs targeting the corresponding transcription factor are underlined.

### CNV-miRNAs can cause Global Impairment of all miRNAs

**Figure 6:**
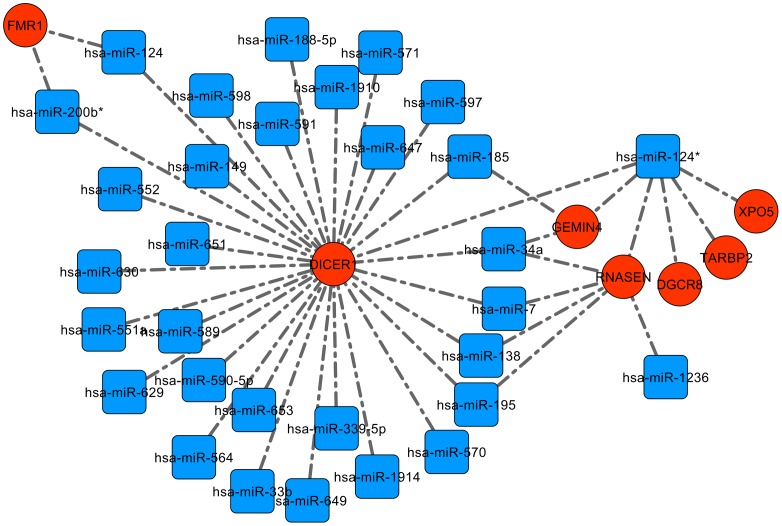
CNV-miRNAs and their target genes involved in the microRNA processing and biogenesis pathways. Saffron ellipses represent the key components involved in miRNA processing and biogenesis, while miRNAs are denoted by the blue coloured squares. Dashed lines represent the validated interactions between the genes and CNV-miRNAs.

While building the data for CNV-miRNA target gene network, we found *DICER1* to be a common and prominent validated target suggesting that the CNV-miRNAs may cause global impairment in the processing of all miRNAs in autistic individuals. Hence we identified the miRNAs which target the key components involved in the biogenesis and processing of miRNAs i.e., *AGO1*, *AGO2*, *DGCR8*, *DICER1*, *FMR1*, *GEMIN3*, *GEMIN4*, *HIWI*, *P68*, *P72*, *RNASEN*, *TARBP2* and *XPO5*, and visualized the interactions using Cytoscape. Further, to test the statistical significance of this network, we considered the interactions of a similar number of random miRNAs with the genes involved in miRNA biogenesis. The CNV-miRNA network was significant in terms of average shortest path length (D = 0.385, *P* = 0.009033), closeness centrality (D = 0.385, *P* = 0.009033), neighbourhood connectivity (D = 0.5789, *P* = 9.93×10^−6^) and radiality (D = 0.7015, *P* = 3.26×10^−8^). Other local properties were identical between the two networks indicating that miRNA biogenesis and processing genes are over studied in disease models. However, one cannot underestimate the clinical relevance since the deregulation of a single or a subset of CNV-miRNAs may have subtle effects on these key components and still may affect the biogenesis and maturation of all miRNAs. It is evident from Figure 6 that *DICER1* has 30 interactions indicating that it is regulated by 30 of the CNV-associated miRNAs while *RNASEN*, *GEMIN4*, and *FMR1* is targeted by at least two different miRNAs. It should be noted that the neuronal miRNA hsa-miR-124* targets multiple genes namely *DICER1*, *RNASEN*, *TARBP2*, *DGCR8* and *XPO5*. Altogether this suggests that variations in the expression of the CNV-miRNAs may affect the global biogenesis and processing of miRNAs in autistic individuals.

## Discussion

Copy number variants and miRNAs are new entities that have changed the order of complexity at the level of gene expression and regulation. Several human studies have established connection between structural chromosomal abnormalities and ASD phenotypes suggesting that rare CNVs collectively contribute to ASD risk (reviewed in [45]). Despite the evidence that ASD is primarily a genetic disorder, it has been extremely difficult to pinpoint the genes and explain the genetic heterogeneity. In addition, many ASD-implicated genes were also associated with other neuropsychiatric disorders, including Schizophrenia, ADHD, Epilepsy, and Intellectual Disability (ID) and none were specific for autism suggesting that additional modifying factors dictate the clinical outcome of having disruptions in specific genes (reviewed in [46]). Research by Hsu et al. (2012) directly implicates miRNAs as functioning in inhibitory synapses and illustrates the global effects imparted by cell-specific knockdown of miRNAs [47]. Also much of our knowledge about individual miRNA functions in autism were primarily derived from miRNA expression profiling in brain tissues and lymphoblastoid cell lines of autistic individuals. We tested whether miRNAs present in CNV loci could potentially explain the less frequent or rare genetic mutations and the genetic heterogeneity underlying ASD. Deletion of a chromosomal loci results in the loss or disruption of the miRNAs while duplication increases the copy number of miRNAs which can have a significant impact on neuronal function and communication since miRNAs provide great control flexibility by integrating signals from different pathways under a variety of physiological conditions. Recently, high-resolution RNA-sequencing of human brain tissues has revealed that miRNA variants (isomiRs) are present in developmental stage and tissue specific profiles [48]. These isomiRs selectively associate with particular argonaute proteins, implying nuanced and context-dependent functional roles. Consistent with this evidence, we observed that isomiRs were also present in the CNV regions. Among the 71 miRNAs, a few including hsa-miR-484, hsa-miR-598, hsa-miR-7, hsa-miR-195, and hsa-miR-211 were previously described to be deregulated in autism [29], [30], [31]. MicroRNA-34a negatively regulates both dendritic outgrowth and synaptic components *Syt-1* and *Stx-1A* [32], [33], although the relevant targets have not yet been confirmed. Further, miR-188 was found to fine tune the levels of its targets, especially *Nrp-2* in response to activity-related plasticity [34]. Hsa-miR-124, a brain enriched miRNA that acts in a temporal and cell specific manner, is known to affect neuronal development, differentiation, neurite branching, and activation of brain microglia (reviewed in [35]). A report by Chi, Hannon and Darnell (2012) presented a new mode for target recognition by hsa-miR-124 that cannot be explained by canonical seed matches [49]. This ‘pivotal’ new rule has expanded the number of potential genes regulated by miRNAs and considering the importance of hsa-miR-124, its deregulation will lead to multiple neuropsychiatric conditions. This gives us insight into miRNAs as a very applicable and exciting avenue for better understanding of neurological diseases and to develop treatment modalities [50].

**Figure 7:**
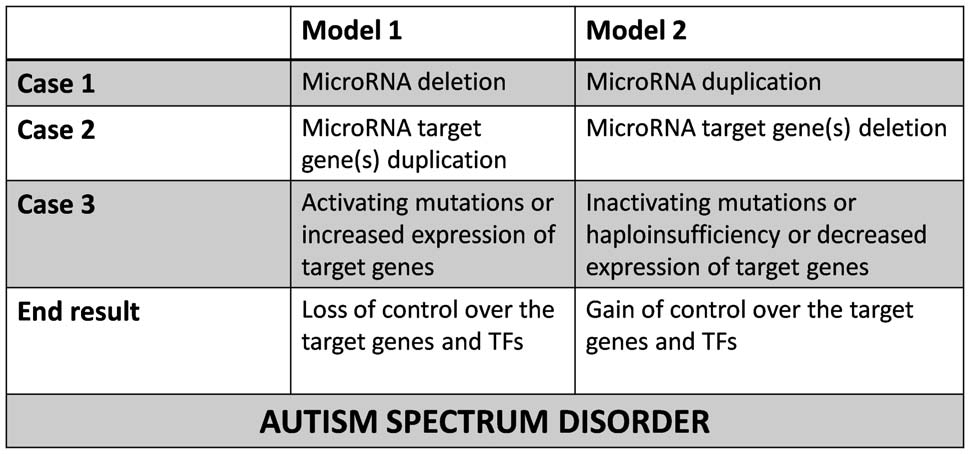
The genetic heterogeneity model of ASD. This figure summarizes the various mechanisms by which the dosage of critical genes involved in autism may be altered thereby contributing to the genetic heterogeneity. To elaborate, individual 1 may harbor CNVs that may cause deletion or duplication of a particular miRNA. In individual 2, the target gene(s) present in the CNV regions might show inverse correlation with the deletion/duplication status of the miRNAs altered in individual 1. In individual 3, some genes may be inactivated by mutation or promoter methylation and certain genes may be over expressed due to loss of miRNA control or increased transcriptional activity. Altered expression of miRNAs may directly affect a transcription factor which in-turn may control the downstream genes, with the phenotypic effect reducing down to those mentioned above.

MicroRNAs need a minimal 2–8 base pair matching with the mRNAs and in this way they can bind to and inhibit the translation of more than 200 mRNAs. MicroRNAs may collaborate by convergence onto key genes or hubs within networks and hence it is essential to “map” all miRNA functions. Differentially expressed miRNAs were found to target genes involved in neurological functions such as *MECP2*, *DISC1*, *NRXN1*, *SHANK2* and *CNTNAP2* [29]. Although initial studies at genomic level suggest that miRNA alterations could contribute to the genetic heterogeneity and phenotypic variation in ASD, miRNA profiling studies have not yet produced a convergent picture suggesting the need for studying miRNA-related pathways. Importantly, research shows that loss of function of *FMRP*, an RNA binding protein, is associated with loss of translational brake on the synthesis of a subset of synaptic proteins which contributes to fragile×syndrome and autism [51]. This study and two other studies [52], [53] highlight the importance of translational regulation in autism, and any perturbations in this process including those caused by miRNAs will lead to cognitive deficits. In addition, direct deletion of the miRNA processing enzyme Dgcr8 in neurons, results in alterations in dendritic branching, excitatory synaptic transmission, and short term plasticity [54], [55], [56]. Of the many miRNAs, hsa-miR-484 and hsa-miR-7-1 targeting *SHANK2* and hsa-miR-218 targeting *NRXN1* were significantly associated with genomic CNVs [57] and differentially expressed in ASD [58]. The results of our gene functional annotation are well in concordance with the enriched GO categories of the ASD gene reference dataset ‘AutRef84’ [59] and reinforce previous hypotheses, such as synaptic biology, transmission of nerve impulse and cognitive deficits. To this end, Levy et al. (2011) conducted network-based analysis of genetic associations (NETBAG) from a list of genes found in autism-associated *de novo* CNVs [60] and reported a preponderance of network genes involved in neuronal motility, targeting of axons and synapse development [61]. Thus miRNAs and their target genes seem to be one of the major players in the development of ASD. Indeed, a rapidly expanding body of evidence suggests that miRNAs and other classes of ncRNAs, along with associated factors, are involved in the pathophysiology of every major class of neurological and psychiatric disorder (reviewed in [62]). The findings of our study can be extended to fit into an overall model that centres on miRNAs and their targets to contribute to the genetic heterogeneity in autistic individuals which mirrors their phenotypic complexity (Figure 7). This might be replicated for other related psychiatric disorders like Schizophrenia as well. Understanding how perturbations in the balance between miRNAs and their target genes lead to disease is crucial in delineating the ASD pathophysiology. As the studies concentrating on the genetic contributions to ASD expand, the notion of miRNAs contributing towards biological convergence can be tested more rigorously. On the whole, it is clear that miRNAs play diverse roles in shaping the neuronal landscape [63].

### Conclusion

MicroRNAs exert precise control over the spatiotemporal expression of individual genes as well as large gene networks, which is crucial for executing complex neurobiological processes.The ability to understand how these tiny post-transcriptional gene regulators function in cellular networks may provide novel therapeutic targets for ASD. Our study investigated the contribution of miRNAs in copy number variable regions towards the development of autism. Approximately 11% of the CNV loci (41 out of 378) were shown to harbor miRNAs. Among the total 71 miRNAs, a few were previously reported to be associated with autism. Analysis of the miRNA–target gene regulatory network has yielded some significant findings and novel insights and has further revealed that miRNAs and their target genes form a tight regulatory backbone. It is clear that miRNAs are components of both the genetic architecture as well as biological pathways that mediate the effects of primary genetic deficits in autism. On the whole, our study provides strong evidence for the role of CNV associated miRNAs in autism and suggests a possible mechanism that will account for the genetic heterogeneity and phenotypic variability of autism in at least a subset of individuals. This knowledge can be translated to identify microRNA biomarkers and to develop therapeutics.

## Materials and Methods

### CNV Datasets and Selection Criteria

CNVs implicated in Autism were retrieved from Autism Database (AutDB) available at http://www.mindspec.org/AutDB.html [26]. This publically available interactive database currently contains the full list of autism susceptibility genes as well as all Copy Number Variations (CNVs) extracted from the studies on molecular genetics and biology of ASD. Further, population information and individual information can be retrieved for each CNV loci from the internal links available in the database. To identify the most important chromosomes that have accumulated several autism-associated CNVs, we calculated the normalized CNV density of each chromosome using the following formula in a step wise manner.
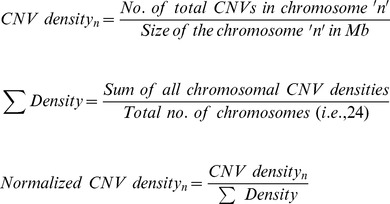



In the above formulae ‘n’ denotes any given human chromosome.

For our *in-silico* analysis, we chose CNVs that were shown to be either only deleted or only duplicated in at least two independent studies. Further, we also chose those CNVs identified by more than 5 different association studies to show both deletion and duplication in different individuals. In total, 378 CNVs found to fit the above mentioned selection criteria were included in our study.

### Gene Datasets

All genes implicated in syndromic and non-syndromic forms of autism were compiled from AutDB and GeneCards (Version 3.08, 20 May 2012) available at http://www.genecards.org/. Results of both these databases were merged after removing the duplicate entries indicating that 708 genes have been associated with autism until now. This served as the master gene list for further comparative analyses.

### Retrieval of CNV-microRNAs and Target Prediction

The miRNAs present in CNVs were identified by analysing the chromosomal coordinates in UCSC Genome Browser (http://genome.ucsc.edu/) utilizing the sno/miRNA prediction tracks of Mar.2006 (NCBI36/hg18) assembly. To understand the function of these miRNAs better, we identified their validated and putative targets from miRWalk database [36] available at http://www.umm.uni-heidelberg.de/apps/zmf/mirwalk/. miRWalk allows the users to choose which combinations of individual source prediction databases they would like to consider when selecting prediction data. For prediction of putative targets by miRWalk, we applied filters set to identify the 3′UTR that has a minimum seed match of 7 nucleotides and retained only the cross-database predicted targets (consistently predicted by miRanda, miRDB, miRWalk and TargetScan). This is because integrating multiple databases improves accuracy or coverage of predictions by balancing out the precision and recall. Data were imported into Microsoft Excel and compared with the list of 708 genes to shortlist those targets associated with autism.

### Network Construction

The autism-associated target genes of the microRNAs present in the CNVs formed the basis of microRNA-mRNA interaction network that was visualized using CytoScape® 2.8.3 available at http://www.cytoscape.org [37]. Considering the complexity of regulatory networks, we computed the degree of each node (measured by the number of links of the node in the network). If the degree distribution of a network follows a power law, the network would have only small portion of nodes with a large number of links (i.e., hub molecules). The top 10 hubs were ranked on the basis of their degree values. The global properties of the network were measured using NetworkAnalyzer [64], a Java plugin for Cytoscape. All the statistical tests were performed using R language and environment (http://www.r-project.org/).

### Functional Annotation of the microRNA Targets

Functional annotation of the microRNA target genes was carried out by **WEB**-based **Ge**ne **S**e**T A**na**L**ysis **T**oolkit (WebGestalt) [65], a public tool that augments and integrates annotations from GO [66]. The official gene symbols of miRNA target genes were uploaded in tab-delimited text format to WebGestalt available at http://bioinfo.vanderbilt.edu/webgestalt. Using the default reference background and classification stringency, GO annotation and KEGG pathway enrichment analysis was performed to derive all the related functions associating with their enrichment scores and p-values, with the multiple test adjustment set to be done by Benjamini & Hochberg. Only the results with a corrected p-value less than 0.05 were considered to be significant.

### Transcription Factor Enrichment Analysis

For the identification of key transcription factors that control the expression of the autism-associated genes targeted by miRNAs, we utilized the Chip Enrichment Analysis tool (ChEA) [67] available at http://amp.pharm.mssm.edu/lib/chea.jsp. The filters were set to identify only those validated human transcription factors that were reported to interact with input gene set by ChIP-Chip (Chromatin immunoprecipitation and microarray) experiments. The miRNAs targeting these TFs were retrieved from miRWalk using the same procedure and criteria described earlier.

## Supporting Information

Table S1 **Details of the 378 autism implicated CNV loci considered for our study with their corresponding microRNA content.**
(XLS)

Table S2 **Functional annotation of the 708 genes associated with autism until now.**
(XLSX)

Table S3 **Functional annotation of all the genes targeted by miRNAs present in the 41 autism-associated CNV loci.**
(XLSX)

Table S4 **The list of genes present in each category of** **Figure 4:** **Intersection analysis of the 326 genes targeted by miRNAs present in CNVs.**
(XLSX)

Table S5 **The list of target genes of the top 10 hub microRNAs identified from the microRNA-target gene network in autism.**
(XLS)
